# Comparison of Ocular Pulse Amplitude Lowering Effects of Preservative-Free Tafluprost and Preservative-Free Dorzolamide-Timolol Fixed Combination Eyedrops

**DOI:** 10.1155/2015/435874

**Published:** 2015-10-18

**Authors:** Du Ri Seo, Seung Joo Ha

**Affiliations:** Department of Ophthalmology, Soonchunhyang University College of Medicine, Soonchunhyang University Seoul Hospital, Seoul 140-743, Republic of Korea

## Abstract

*Purpose*. To compare the ocular pulse amplitude (OPA) lowering effects of preservative-free tafluprost and dorzolamide-timolol fixed combination (DTFC) using dynamic contour tonometry. *Methods*. In total, 66 eyes of 66 patients with normal tension glaucoma (NTG) (*n* = 34) or primary open angle glaucoma (POAG) (*n* = 32) were included. Patients were divided into two groups: the preservative-free tafluprost-treated group (*n* = 33) and the preservative-free DTFC-treated group (*n* = 33). Intraocular pressure (IOP) was measured using Goldmann applanation tonometry (GAT). OPA was measured using dynamic contour tonometry; corrected OPA (cOPA) was calculated at baseline and at 1 week and 1, 3, and 6 months after treatment. *Results*. After 6 months of treatment, tafluprost significantly reduced IOP (*P* < 0.001). The OPA lowering effects differed significantly between the two treatment groups (*P* = 0.003). The cOPA-lowering effect of tafluprost (1.09 mmHg) was significantly greater than that of DTFC (0.36 mmHg) after 6 months of treatment (*P* = 0.01). *Conclusions*. Tafluprost and DTFC glaucoma treatments provided marked OPA and IOP lowering effects. Tafluprost had a greater effect than DTFC; thus, this drug is recommended for patients at risk of glaucoma progression, due to the high OPA caused by large fluctuations in IOP.

## 1. Introduction

Glaucoma describes a group of ocular disorders of multifactorial etiology, united by a clinically characteristic optic neuropathy, with potentially progressive, clinically visible changes at the optic nerve head corresponding to diffuse and localized nerve-fiber-bundle pattern visual field loss [1]. Risk factors for the development and progression of glaucoma include elevated intraocular pressure (IOP), decreased ocular perfusion pressure, older age, thinner central corneal thickness, and disc hemorrhage [2, 3]. Elevated IOP is the main risk factor for glaucoma development and progression; thus, to date, IOP reduction is the only well-documented, successful glaucoma treatment [4]. However, glaucoma also develops in individuals who have never experienced elevated IOP; moreover, in some patients, glaucoma can progress even when IOP reaches the target level.

Previous studies suggest that ocular pulse amplitude (OPA) may play a role in the clinical course of glaucoma. OPA is derived from the difference between the maximum and minimum IOP during an IOP measurement session [5]. OPA is believed to be caused by the blood volume that is pumped into the eye (mainly, the choroidal bed) during each cardiac cycle. Therefore, OPA may reflect volumetric changes that are dependent on ocular blood flow [6]. Izumi et al. [7] reported that the administration of 0.0015% tafluprost significantly increased retinal blood flow and blood velocity, as determined by laser Doppler velocity measurements in cats. Dorzolamide-timolol fixed combination (DTFC) also improves retinal blood flow to some extent [8]. Glaucoma management requires long-term and sometimes lifelong treatment. Benzalkonium chloride (BAK), which is widely used in glaucoma preparations as a preservative, damages the tear film and has a negative impact on the number of conjunctival goblet cells. For this reason, over 50% of patients treated for glaucoma have concurrent ocular surface disorders [9].

Recently, a preservative-free antiglaucoma drug has been made available. Studies have shown that the IOP reduction obtained with the use of the preservative-free formulation is equivalent to that achieved by the preserved formulation [10, 11]. Representative preservative-free antiglaucoma drugs include tafluprost, the first preservative-free formulation among prostaglandin analogues, and DTFC.

The present study was conducted to compare the effects of preservative-free tafluprost and preservative-free DTFC on IOP and OPA in patients with primary open angle glaucoma (POAG) and normal tension glaucoma (NTG).

## 2. Material and Methods

This investigation is a retrospective analysis of 80 patients, newly diagnosed with POAG or NTG and treated with preservative-free tafluprost or preservative-free DTFC, who were enrolled from a clinical database at the glaucoma clinic at Soonchunhyang University Hospital (Seoul, Korea) between September 2013 and August 2014. The study was conducted in accordance with the ethical principles of the Declaration of Helsinki and was approved by the Institutional Review Board of Soonchunhyang University Hospital.

Upon initial examination, each participant underwent a comprehensive evaluation, which included a detailed review of their ocular and medical histories, measurement of visual acuity, central corneal thickness measurement using ultrasound pachymetry (Tomey Corporation, Nagoya, Japan), gonioscopic examination, cup-to-disc ratio measurement by fundus photography (VX-10, Kowa Optimed, Tokyo, Japan), measurement of the retinal nerve fiber layer thickness by optical coherence tomography (OCT, Heidelberg Engineering, Heidelberg, Germany), IOP measurement by Goldmann applanation tonometry (GAT), dynamic contour tonometry (DCT, PASCAL, Swiss Microtechnology AG, Port, Switzerland), OPA measurement by DCT, and automated perimetry using the 24-2 Swedish Interactive Threshold Algorithm standard program (Humphrey Visual Field Analyzer, Carl Zeiss Meditec, Dublin, CA, USA).

Glaucoma was defined by the presence of a characteristic glaucomatous disc and retinal changes associated with typical, reproducible visual field (VF) defects on standard automated perimetry. An abnormal VF was defined as a Glaucoma Hemifield Test result outside the normal limits on at least two consecutive VF tests and a cluster of three or more contiguous nonedge points on pattern deviation probability plots (with a probability of less than 5%) with at least one of these points with a probability of less than 1%. The VF tests required reliability indices better than 25% to be included. A maximum IOP of untreated 21 mmHg or more, as indicated in Goldmann tonometry measurements, was required for a diagnosis of POAG. A maximum untreated IOP of 21 mmHg or less was required for a diagnosis of NTG.

Due to their potential effects on IOP, systemic drugs were not administered to any of the patients. In cases in which both eyes were eligible for the study, one was randomly chosen for inclusions. From the 80 patients, individuals with a corneal disorder that could interfere with optimal GAT or DCT, a history of ocular surgery or trauma, or evidence of ocular infection or those who had a laser procedure on the eye were excluded from the study. Eventually 66 eyes of 66 patients were included.

IOP and OPA were measured using GAT and DCT, respectively, before treatment and after 1 week and 1, 3, and 6 months of treatment, at the same time of day for each patient, without additional treatments such as additional drugs, laser procedures, or ocular surgery. The quality of the DCT measurement ranged from 1 to 5. Good reliability measurements (*Q* ≤ 3) were included in this study. The corrected OPA (cOPA) was calculated to determine the pure value of the OPA, excluding the influence of IOP. The cOPA formula is given by the following: cOPA = OPA − (ΔIOP × 0.12) [12, 13]. The Mann-Whitney *U*-test was used to compare the IOP, OPA, and cOPA of the two groups. Wilcoxon's signed rank test was used to evaluate changes in IOP, OPA, and cOPA in the two groups. Bonferroni correction was used to adjust *P* values. All of the analyses were conducted using SPSS version 18.0 (SPSS Inc., Chicago, IL). In all of the analyses, *P* < 0.05 indicated statistical significance.

## 3. Results

In total, 66 eyes of 66 subjects were included in this study: 33 eyes in the tafluprost group and 33 eyes in the DTFC group. In the tafluprost group, 9 eyes were diagnosed with NTG and 24 eyes presented with POAG. In the DTFC group, 25 eyes presented with NTG and 8 eyes indicated POAG. Between the tafluprost and DTFC groups, no significant difference was evident regarding age, cup-to-disc ratio, central corneal thickness, visual field mean deviation, or the thickness of circumpapillary retinal nerve fiber layer. However, the IOP of the tafluprost group was higher and was statistically significant (*P* < 0.001) due to the greater number of POAG patients in this group (Table 1).

**Figure 1:**
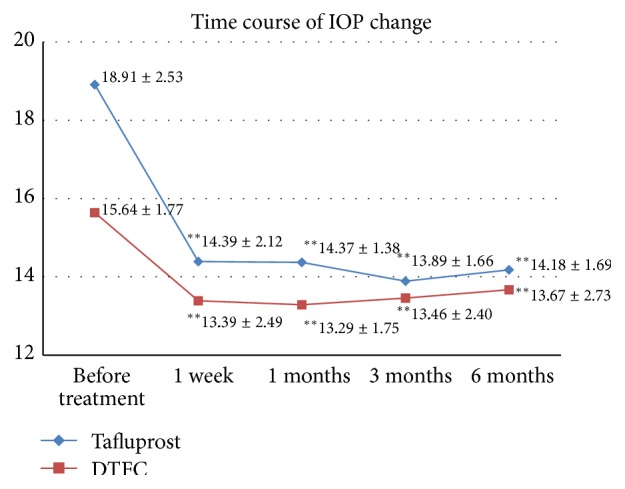
Change in intraocular pressure (IOP) (mean ± SD) in tafluprost and dorzolamide-timolol fixed combination (DTFC) treatment groups. ^*∗∗*^Statistically significant (*P* < 0.05, Mann-Whitney *U*-test,* P* value by Bonferroni correction).

The mean IOP findings for baseline and at 1 week and 1, 3, and 6 months were 18.91 ± 2.53, 14.39 ± 2.12, 14.37 ± 1.38, 13.89 ± 1.66, and 14.18 ± 1.69 mmHg, respectively, for the tafluprost-treated group, and 15.64 ± 1.77, 13.39 ± 2.49, 13.29 ± 1.75, 13.46 ± 2.40, and 13.67 ± 2.73 mmHg, respectively, for the DTFC-treated group. At 6 months, the mean IOP reduction from the baseline value was −4.73 mmHg (25.0%) for the tafluprost group compared to −1.97 mmHg (12.6%) for the DTFC group. The difference in IOP reduction between the two groups was statistically significant (*P* < 0.001) (Figure 1).

**Figure 2:**
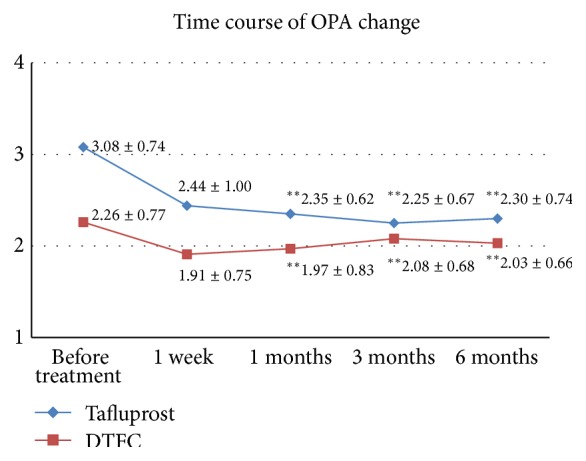
Change in the ocular pulse amplitude (OPA) (mean ± SD) in the tafluprost and DTFC treatment groups. ^*∗∗*^Statistically significant (*P* < 0.05, Mann-Whitney *U*-test,* P* value by Bonferroni correction).

The mean OPA for baseline and at 1 week and 1, 3, and 6 months was 3.08 ± 0.74, 2.44 ± 1.00, 2.35 ± 0.62, 2.25 ± 0.67, and 2.30 ± 0.74 mmHg, respectively, for the tafluprost-treated group, and 2.26 ± 0.77, 1.91 ± 0.75, 1.97 ± 0.83, 2.08 ± 0.68, and 2.03 ± 0.66 mmHg, respectively, for the DTFC-treated group. At 6 months, the mean decrease in OPA from the baseline value in the tafluprost group was −0.78 mmHg (25.3%) compared to −0.23 mmHg (10.2%) for the DTFC group. The OPA-reducing effect was stronger in the tafluprost group than in the DTFC group. The difference in OPA between the two groups was statistically significant (*P* = 0.003) (Figure 2). However, OPA is reported to be positively correlated with IOP and the OPA-reducing effect of tafluprost is affected by IOP at baseline [14, 15]. Therefore, the cOPA, a value of OPA adjusted to exclude the influence of IOP, was compared.

**Figure 3:**
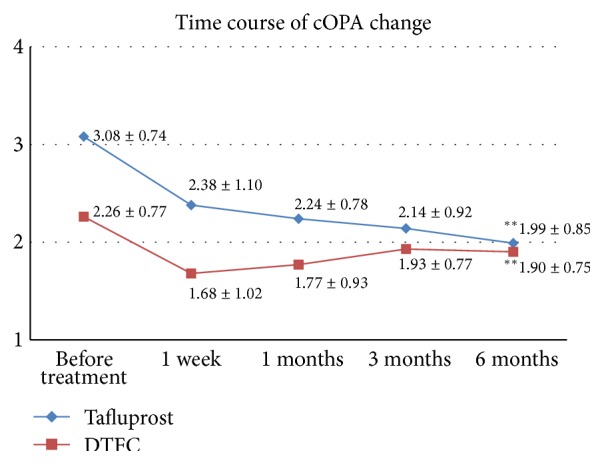
Change in the corrected ocular pulse amplitude (cOPA) (mean ± SD) in the tafluprost and DTFC treatment groups. ^*∗∗*^Statistically significant (*P* < 0.05, Mann-Whitney *U*-test,* P* value by Bonferroni correction).

In the tafluprost group, the mean cOPA was 3.08 ± 0.74, 2.38 ± 1.10, 2.24 ± 0.78, 2.14 ± 0.92, and 1.99 ± 0.85 mmHg for baseline and after 1 week and 1, 3, and 6 months, respectively. In the DTFC group, the mean cOPA was 2.26 ± 0.77, 1.68 ± 1.02, 1.77 ± 0.93, 1.93 ± 0.77, and 1.90 ± 0.75 mmHg, respectively. At 6 months, the mean cOPA from the baseline value decreased by −1.09 mmHg (35.4%) for the tafluprost group and −0.36 mmHg (15.9%) for the DTFC group. The cOPA decreased gradually in the tafluprost group over the follow-up period. This difference in cOPA between the two groups was statistically significant (*P* = 0.01) (Figure 3).

## 4. Discussion

Many studies have attempted to identify and measure ocular perfusion abnormalities, reportedly a risk factor for the occurrence and progression of glaucoma [6, 10, 16]. Pulsating ocular perfusion, known to mainly reflect choroidal perfusion, is calculated by measuring the OPA, which is thought to be caused by the blood volume that is pumped into the eye during each cardiac cycle. Therefore, OPA is an indirect measurement of pulsating ocular perfusion [17]. DCT, used in this study, represents a novel type of recording tonometry giving a reading of IOP and OPA [12]. According to the working principles of DCT, matching up the concave pressure sensor with cornea provides direct measurements independently of corneal properties [18].

DTFC and tafluprost, both topical antiglaucoma drugs, improve ocular perfusion [8, 19]. Differences in the change in OPA between the two treatment groups in this study began to appear after 1 month of drug administration. OPA usually decreases in proportion to IOP lowering; thus, the effect of IOP reduction must also be taken into account to analyze OPA change. The corrected OPA values were significantly different after 6 months of drug administration in our study. However, the fact that the baseline IOP was higher in the tafluprost group may be a limitation in interpreting the results.

Jang et al. [20] evaluated the differences in the IOP and OPA in patients who were only treated with DTFC or latanoprost. The decrease in IOP was not significantly different between the two groups. The OPA showed no change in the DTFC group; however, a significant decrease in OPA was observed for the latanoprost group.

According to the above results, prostaglandin drugs are thought to have a bigger effect on the decrease in OPA than other drugs. Our study correlates with previous studies by showing that tafluprost decreases the OPA more than DTFC. The role of OPA in the etiology and progression of glaucoma is yet unclear. Although some claim that low OPA is a representative marker for the progression of glaucoma and visual damage [21, 22], high OPA, based on theoretical mechanism, indicates that the change in the ocular pressure fluctuates to a greater degree each cardiac cycle and may present an additional risk factor for glaucoma progression. On the basis of the published data, tafluprost directly relaxes the retinal microvasculature and/or the retrobulbar arteries [23]. Several studies have indicated that DTFC therapy increases perfusion to the retinal capillaries and potentially oxygen delivery to retinal tissues [8, 19]. Because OPA may reflect volumetric changes that depend on ocular blood flow, decreasing OPA in this study may reflect a constantly increasing retinal blood flow.

Long-term administration of ocular drugs containing preservatives may cause discomfort in glaucoma patients; moreover, as the number of drugs increases, the frequency of ocular surface disease also increases. Preservative-free antiglaucoma drugs reportedly improve both objective and subjective symptoms in patients and their quality of life [24, 25]; they also exhibit similar effectiveness as the drugs containing preservatives [11, 26]. The second aim of our study was to determine if preservative-free and preservative-containing drugs have the same effects on lowering IOP and OPA. Our results showed that the preservative-free drugs had similar effects to that of preservative-containing drugs in decreasing the ocular pressure and OPA.

The limitations of this study included the small sampling of patients. No crossmatched study involving the same group of patients was performed. In addition, data were not available on the axial length of the eye; this information would have been particularly useful, because OPA is negatively correlated with the axial length [27]. In addition, the initial ocular pressure in the two groups was different and may have confounded the interpretation of the results. The comparatively high number of POAG patients in the tafluprost group (24 eyes compared to 9 presenting NTG) was inevitable, with the study performed under certain clinical settings; this is because tafluprost is well known for its strong IOP lowering effect on POAG patients [28, 29]. However, cOPA analysis may supplement these limitations. Finally, this study shows not only the difference of IOP and OPA reduction but also the difference between the two drugs' effects on cOPA reduction and pure OPA reduction excluding the effect of baseline IOP which enhances the effects of tafluprost to a greater extent than those of DTFC. This study also shows that both drugs lower cOPA, in line with tafluprost's mechanism; it is a new potent prostaglandin analog with high affinity for the fluoroprostaglandin receptor (PGF2*α*). Tafluprost inhibits endothelial-1-induced impairment of optic nerve head blood flow, and its effect is longer than travoprost and latanoprost in rabbit eyes [30]. In a follow-up, different drugs' effect on cOPA in the same IOP groups will be studied to compensate for the limitations of this study.

## 5. Conclusions

Treatment with preservative-free tafluprost and preservative-free DTFC resulted in a significant reduction in IOP and OPA and can be used in glaucoma patients who are sensitive to preservatives. When considering tafluprost for OPA reduction, our results indicate that it will be most effective for patients who exhibit well-controlled IOP but continue to show glaucomatous changes or high OPA.

## Figures and Tables

**Table 1:** Patient characteristics in the tafluprost and DTFC treatment groups.

	Tafluprost (33 eyes of 33 patients)	DTFC (33 eyes of 33 patients)	*P* value
Mean age ± SD (years)	53.6 ± 13.5	54.4 ± 12.2	0.768
Sex (male : female)	17 : 16	16 : 17	0.625
SE (diopter)	−2.9 ± 4.3	−2.4 ± 2.7	0.533
Diagnosis (NTG : POAG)	9 : 24	25 : 8	<0.001
CCT ± SD (*μ*m)	534.6 ± 33.9	545.2 ± 38.3	0.199
Cup-to-disc ratio	0.6 ± 0.2	0.6 ± 0.2	0.853
Pretreatment IOP (mmHg)	18.91 ± 2.53	15.64 ± 1.77	<0.001
Posttreatment IOP (mmHg)	14.18 ± 1.69	13.67 ± 2.73	0.338
IOP reduction (%)	25.0	12.6	
Pretreatment OPA (mmHg)	3.08 ± 0.74	2.26 ± 0.77	<0.001
Posttreatment OPA (mmHg)	2.30 ± 0.74	2.03 ± 0.66	0.192
OPA reduction (%)	25.3	10.2	
Pretreatment cOPA (mmHg)	3.08 ± 0.74	2.26 ± 0.77	<0.001
Posttreatment cOPA (mmHg)	1.99 ± 0.85	1.90 ± 0.75	0.748
cOPA reduction (%)	35.4	15.9	
Baseline VF MD (decibel)	−9.4 ± 7.6	−9.1 ± 5.7	0.909
OCT cpRNFL thickness (micron)	75.7 ± 17.2	76.4 ± 15.4	0.577

NTG: normal tension glaucoma; POAG: primary open angle glaucoma; SE: spherical equivalent; CCT: central corneal thickness; IOP: intraocular pressure; OPA: ocular pulse amplitude; cOPA: corrected OPA; VF MD: visual field mean deviation; OCT: optical coherence tomography; cpRNFL: circumpapillary retinal nerve fiber layer.
